# Internal jugular vein compression: A benign entity or an underappreciated phenomenon?

**DOI:** 10.1016/j.ensci.2025.100577

**Published:** 2025-07-22

**Authors:** Paul Francis Greene

**Affiliations:** Medical College of Wisconsin, Milwaukee, WI, USA

The pendulum may swing wildly during progress of science and medical understanding. Caution and critical analysis are prudent, not only to avoid rushing hastily ahead, but also to ensure promising momentum is not abandoned.

General venous pathology in the body, including chronic venous insufficiency, has been identified as a strong predictor of all-cause death [hazard ration (HR) 1.46 (95 % confidence interval (CI) 1.19–1.79) *P* = .003] and associated with an increased risk of all-cause death [HR 1.51 (95 % CI 1.11–2.05) *P* = .009] [1]. If the altered return of blood from the body can presumably be pathologic, one may inquire what the result of altered venous drainage from the brain may be. Even though the waste clearance mechanism of the body's venous system is not well understood, venous outflow, especially from the brain, may be critical to function [2].

Cerebral venous compression, congestion, stenosis, and/or insufficiency can encompass an umbrella of entities; however, for this letter to the editor the primary focus will be at the internal jugular vein (IJV). Drainage by the IJV may be disrupted by thrombosis, vein wall pathology and/or various extrinsic compressions with narrowing occurring at different levels, though often between the transverse process or arch of C1 and the styloid process [3]. An elongated styloid process causing impingement is defined as Eagle syndrome [4,5], yet other extrinsic structures may also compress the IJV along with other potential neurovascular in the region [[6], [7], [8]].

In 2012, Jayaraman et al. concluded that extrinsic compression, specifically at the superior segment of the IJV, is a common finding. Of note, in this retrospective study of computed tomography (CT) angiograms, the 108 patients were reported to have a “neurologic reason” for the test, and the authors revealed that this series of patients could be different than a control population of normal subjects [9]. Personally, I am interested in additional details about the subjects included, such as baseline demographics and symptoms, if any. Without normal controls, to me it appears a bit premature to have concluded extrinsic compression was unlikely to be pathologic in nature [9].

Subsequent assessment of CT scans from 500 patients, completed due to a wide range of indications, demonstrated 50 % narrowing of the IJV in 36.6 % of the patients at various levels, but most frequently at C1 [10]. Occlusion or near-occlusion of the superior IJV was also observed in approximately one-quarter of the 164 patients undergoing CT angiography [11]. More recently, an analysis of 1487 patients with contrast enhanced magnetic resonance (MR) venograms and MR imaging, compared those with or without positive symptoms to those with or without IJV stenosis, and suggested it may be feasible to distinguish between physiologic and pathological IJV stenosis [12]. The authors advised to consider clinical symptoms when differentiating between physiological IJV slenderness and pathological IJV stenosis with the latter including features such as cloudy-like white matter hyper-intensities (WMHs) and surrounding abnormal venous collaterals [12]. Similarly, patients with idiopathic intracranial hypertension had statistically significant greater flow in extrajugular networks compared to controls [13]. Nonetheless, future prospective studies have been recommended to better comprehend different imaging modalities and the significance of IJV stenosis [14].

Paolo Zamboni's research, published in 2009, sent tidal waves across the multiple sclerosis (MS) world after reporting his results from endovascular treatment of chronic cerebrospinal venous insufficiency (CCSVI) in MS patients [15]. Percutaneous transluminal angioplasties (PTAs) were performed at the levels of the IJV and azygous vein with reported positive results at 18 months in the 65 MS patients [35 with relapse remitting (RR), 20 with secondary progressive (SP), and 10 with primary progressive (PP)] with decreased postoperative venous pressure (*P* < .001) which coincided with improvements in MS clinical outcome measures [rate of relapse-free patients (P < .001) and MR Gad+ lesions (*P* < .0001) in RR group, improved MS Functional Composite at 1 year in RR group (*P* < .008) but not PP or SP groups, Physical Quality of Life (QOL) improved in RR (*P* < .01) and PP (*P* < .03) groups and positive trend in SP (*P* < .08) and Mental QOL improved in RR (*P* < .003) and PP (P < .01) but not SP]. [15]

Critics quickly pointed out shortcomings of Zamboni's work with Requarth (2010) quoting the procedure to have “created an unfortunate urban myth that desperate MS sufferers will cling to” and reporting medical therapy provided lower relapse rates than those treated with angioplasties [16]. In response to calls from the National Institute for Health and Care Excellence and the Italian Ministry of Health along with public urging, Zamboni completed a randomized clinical trial published in 2018 which concluded venous PTA, though safe, was largely ineffective [17]. This trial, the Brave Dreams trial, 2018 included 115 RR MS patients with 76 in the PTA group and 39 to the sham group [17]. Specifically there was no difference between the PTA and sham groups in the functional composite measure [41.7 % vs 48.7 %; odds ratio, 0.75; 95 % CI, 0.34–1.68; *P* = .49], in number of combined lesions on magnetic resonance imaging (MRI) at 6 to 12 months mean 0.47 [standard deviation (SD) 1.19] vs 1.27 [2.65] [mean ratio, 0.37; 95 % CI, 0.15–0.91; *P* = .03: adjusted *P* = .09] or 0 to 12 months 1.40 [4.21] vs 1.95 [3.73] [mean ratio, 0.72; 95 % CI, 0.32–1.63; *P* = .45; adjusted P = .45], in percent free of new lesions on MRI follow-up after 6 to 12 months, 58 of 70 patients (83 %) vs 22 of 33 (67 %) [odds ratio, 2.64; 95 % CI, 1.11–6.28; *P* = .03; adjusted *P* = .09] or at 0 to 12 months, 46 of 73 patients (63.0 %) vs. 18 of 37 (49 %) [odds ratio, 1.80; 95 % CI, 0.81–4.01; *P* = .15; adjusted *P* = .30]. [17]

It is interesting to note in the Zamboni et al. (2018) study that at 12 months blinded flow assessment using doppler ultrasonography demonstrated restored flow in 38 of 71 patients (54 %) in the intervention group with PTA at the level of the IJV and azygos vein compared to in 14 of 37 (38 %) in the sham group [17]. In conclusion, it was broached that a subgroup of MS patients may possibly benefit from PTA and should be further analyzed and investigated [17]. I am curious of the clinical course in those who had restored flow compared to those who did not. Moreover, does the location of compression matter considering fluid dynamic modeling suggesting that compression in the upper parts of the IJV may be more likely to cause pathology by altering outflow from the brain? [18]

Ferral (2020) detailed the “explosive phenomenon” on social media forums and MS support groups following Zamboni's initial study in 2009, the subsequent heated debate, and the Food and Drug Administration's warning letter in 2012 recommending against performing the coined “liberation” procedure. Even despite negative press, anecdotal observation was highlighted, demonstrating that certain patients did benefit from those aforementioned angioplasty interventions [19]. Expanded analysis of the Brave Dreams data demonstrated venoplasty decreased new cerebral lesions at 1 year compared to sham [RR 1.42; 95 % CI 1.00–2.10; *P* = .032] and specifically patients with favorable venograms had a significant higher probability of being free of new lesions compared to patients with unfavorable venograms [RR 1.82; 95 % CI 1.17–2.83; *P* = .005] or patients in the sham group [RR 1.66; 95 % CI 1.16–2.37, P = .005]. [20]

Many recent advancements have been outside the context of MS. Jayaraman et al. (2012) had performed a National Library of Medicine (PubMed) search with the terms “extrinsic compression internal jugular vein” through August 16, 2011. As of March 28, 2025, those same search terms only yielded 21 results compared to the 5 Jayaraman et al. reported before August 16, 2011. However, when broadening the search to the not-as-selective “jugular compression” 945 results were procured including 471 from 2011 and prior. All these results were not pertinent, but nonetheless captured additional studies to give a more complete understanding of the phenomenon. PubMed searches of “jugular” with “stenosis,” “congestion,” and “insufficiency” were also completed. References of articles selected for inclusion were then also scanned for a thorough survey.

Compression of the IJV has been reported to be associated with intracranial hypertension [[3], [10], [21], [22], [23], [24], [25], [26], [27], [28], [29], [30], [31], [32], [33], [34], [35], [36], [37], [38], [39], [40], [41], [42]], headache [3,[24], [25], [26], [27], [28], [29],^31^,[33], [34],[36], [37], [38], [39],[41], [42], [43], [44], [45], [46], [47], [48], [49], [50], [51], [52], [53]], tinnitus [3,23,26,27,29,31,34,39,42,44,46,47,49,51,52,54], dizziness [3,26,27,34,[42], [43], [44], [45], [46],^49^,^51^,^53^], eye symptoms including pressure and blurring [3,23,27,29,31,34,36,37,[41], [42], [43], [44],^46^,^47^,^49^,^51^,^52^], vertigo [[3], [23], [49]], memory disturbances [44,47,49,50], insomnia [3,27,29,31,44,46,47,49], head noises [31,46,47,49], neck discomfort [3,29,44,[47], [48], [49],^55^], ear pain [55], hearing impairment [3,44,46,47,49], cognitive fatigue and brain fog [34], anxiety or depression [44,47,49], papilledema [3,36,42,43,52], thrombosis [36,40,41,50,[56], [57], [58], [59]], and cranial hemorrhage [41,60]. Additional compression in the same region has reported to involve the hypoglossal nerve causing tongue weakness and fasciculation [6], while the glossopharyngeal nerve, vagus nerve, and spinal accessory may also be impinged [61]. Admittedly, there is the potential that some of these non-specific symptoms, such as headache, may be misattributed to IJV compression by the so-called nocebo effect [62]. Regardless I do not feel collectively these reported symptoms can be ignored, and clinical suspicion should aid the provider when examining the global picture.

Dating back to 1928 it was observed that compression of the jugular veins caused ocular hypertension [63]. The understanding of the effect on the brain from IJV compression is continuing to evolve. In mouse studies, cerebral venous congestion triggered blood-brain barrier disruptions and neuroinflammation [64]. Likewise, mice with jugular vein ligations were found to have more cerebral microhemorrhages than controls [65]. The central nervous system in rats, who also have their jugular veins ligated, demonstrated homeostatic disturbances with increased neural metabolic activity, ultrastructural synaptic changes, upregulated metabolic activity, reduced inhibitory synaptic transmission, and elevated expression of neuroplastic related proteins [66]. As aforementioned, humans with pathologic IJV stenosis were observed to have cloudy-like white matter hyper-intensities [12], and Zhou et al. (2019) observed bilateral demyelination changes in the periventricular area and/or centrum semiovale in 95.3 % (41/43) of patients along with single-photon emission CT demonstrating reduced cerebral perfusion and increased cerebral glucose consumption in 92.3 % (24/26) of patients with IJV stenosis [44].

Treatment of IJV compression has consisted of anticoagulants, endovascular procedures, surgery, or a combination [3]. A review of 13 articles consisting of 149 patients demonstrated an improvement in 72.5 % (58/80) of patients with a complication rate of 23 % [3]. Likewise, a literature search through December 2020 yielded 17 studies which were reviewed with styloidectomy yielding 79 % success rate compared to 66 % for angioplasty/stenting without complications reported [35]. Specifically looking at IJV stenting and long term follow up (reported as 30.5 +/− for overall group including the cerebral venous sinus stenosis group) a statistically significant reduction in headache continued with 4 patients compared to 14 patients at baseline reporting headache (*P* < .01) out of 27 patients total in the group with overall complication rate of 7 % (2 out of 27) [67]. It has been advised that neurointerventionalists have experience before performing IJV stenting for a greater understanding of stent size selection, associated anatomy, and patient selection as although the procedures are promising there is the risk of a high complication rate and the return of symptoms [68].

The aim of this article is to present IJV compression in a new light. It is possible the entity of IJV compression has been underappreciated along with the subsequent ramifications. I encourage you to follow the science with an open mind which may not lead to a miracle cure, but nonetheless will likely provide more answers along with more questions. I feel science is the word of reason and has the strength to withstand outside pressures. Science must always move forward, though undoubtedly will have some wild swings from time-to-time.

Cerebral venous outflow from the brain is not fully understood. IJV compression initially was implied to be benign; however, recent advancements indicate in select patients IJV compression, or perhaps better restated as stenosis in these cases, can be pathological. Evidence is emerging that the presence of surrounding venous collaterals and WMHs may assist in distinguishing if IJV compression is benign or pathological. A wide range of non-specific symptoms have been reported to be associated with IJV compression. There appears to be a need for continued awareness of IJV compression, greater understanding of the pathological mechanisms, and additional development in identifying which patients would benefit from treatment.

## CRediT authorship contribution statement

**Paul Francis Greene:** Writing – original draft, Resources, Conceptualization, Writing – review & editing.

## Declaration of competing interest

No potential conflict of interest was reported by the author(s).
